# Cluster of Nasal Rhinosporidiosis, Eastern Province, Rwanda

**DOI:** 10.3201/eid2509.190021

**Published:** 2019-09

**Authors:** Annie I. Izimukwiye, Djibril Mbarushimana, Marie C. Ndayisaba, Venerand Bigirimana, Belson Rugwizangoga, Alvaro C. Laga

**Affiliations:** University Central Hospital of Kigali, Kigali, Rwanda (A.I. Izimukwiye, D. Mbarushimana, M.C. Ndayisaba, V. Bigirimana, B. Rugwizangoga);; Brigham and Women’s Hospital and Harvard Medical School, Boston, Massachusetts, USA (A.C. Laga)

**Keywords:** nasal rhinosporidiosis, Rwanda, rhinosporidiosis, Rhinosporidium seeberi, mesomycetazoan, respiratory infections

## Abstract

We report 4 recent cases of nasal rhinosporidiosis in Rwanda. All patients were boys or young men living in the same district (Gatsibo District, Eastern Province), suggesting a reservoir in the area. The recent reemergence of rhinosporidiosis in Rwanda might reflect increased availability of diagnostic services rather than emerging disease.

Rhinosporidiosis is a tropical disease of the mucous membranes (and, in rare cases, the skin or internal organs) caused by *Rhinosporidium seeberi*, an enigmatic microorganism of disputed taxonomy (*1**–**3*). *R. seeberi* has not been isolated from the environment and has no known natural host or reservoir; consequently, it has been difficult to classify. Originally considered to be a protozoan and subsequently a fungus, *R. seeberi* is currently classified as an aquatic mesomycetazoan (“between fungi and animals”), on the basis of phylogenetic analysis of 18S rDNA (*1**–**3*). 

Originally described in Argentina in 1900 (*4*), rhinosporidiosis is most prevalent in India and Sri Lanka, followed by countries in South America and Africa (*5*). In Africa, rhinosporidiosis has been documented in Cameroon, Democratic Republic of the Congo, Côte d’Ivoire, Kenya, Malawi, South Africa, Tanzania, Uganda, and Zambia, predominantly as conjunctival disease (*6**–**12*). One previous report documented 3 cases of rhinosporidiosis in Rwanda: 2 in the nose (in a 60-year-old man in 1975 and in a 16-year-old young woman in 1977) and 1 in the conjunctiva (in a 3-year-old boy in 1986) (*13*). We report 4 recent cases of nasal rhinosporidiosis diagnosed by using histopathologic methods at the University Central Hospital of Kigali (CHUK) in Kigali, Rwanda.

## The Study

We conducted a retrospective search for the period 2016–2013. We confirmed histopathologic diagnosis by identification of characteristic structures (e.g., sporangia containing endoconidia) on hematoxylin and eosin–stained sections. For 2 cases, we obtained periodic acid–Schiff and Grocott methenamine silver stains for further characterization. For each case, patient age, sex, place of residence, clinical signs and symptoms, and year of diagnosis were recorded.

We identified 4 cases of rhinosporidiosis (Table). The diagnosis was not suspected on clinical grounds. In keeping with previous studies, all patients were young boys and teenagers (7–15 years of age) who had nasal obstruction and a friable mass that bled on touch. Symptoms lasted from 2 months to 4 years (median 8.5 months) and consisted of epistaxis and sensation of mass but no discharge, pain, or pruritus. (*8**,**10**,**11**,**13*). All patients were treated by excisional biopsy, and the diagnosis was made by using histopathologic methods. All 4 patients received nonsteroidal antiinflammatory drugs for symptomatic relief before and after surgery but not antimicrobial therapy. After an early recurrence treated by reexcision (case 3 [Table]), all patients were free of disease at 36 months after excision. All patients lived in the same region (Eastern Province) of Rwanda, and 3 of them lived in the same village (in Gatsibo District). Two cases were diagnosed in 2014, 1 in 2015, and 1 in 2016. No additional cases have been documented since.

**Table:** Clinical summary of 4 patients with rhinosporidiosis, Eastern Province, Rwanda, 2014–2016

Case no.	Patient age, y/sex	Signs and symptoms	District of residence	Method of diagnosis	Year of diagnosis	Treatment and outcome
1	7/M	Nasal bleeding and obstruction for 5 mo; reddish mass on right nasal cavity, adherent to lateral wall; bleeding on touch	Gatsibo	Punch biopsy	2014	Excision; no recurrence at last follow-up (4 y)
2	15/M	Nasal blockage with sensation of nasal mass for 2 mo; vascularized mass; bleeding on touch	Gatsibo	Excisional biopsy	2014	Excision; no recurrence at last follow-up (4 y)
3	13/M	Nasal blockage for 12 mo; mobile, expansile, nontender mass on right nostril; bleeding on touch	Gatsibo	Punch biopsy	2015	Excision; recurrence 3 mo after diagnosis; reexcision with no recurrence at last follow-up (3.5 y)
4	12/M	Progressive nasal blockage for 4 y; soft mass in left nostril attached to the septum; bleeding on touch	Kirehe	Excisional biopsy	2016	Excision; no recurrence at last follow up (3 y)

All biopsies showed numerous sporangia (juvenile, immature, and mature forms) and prominent lymphoplasmacytic inflammation with eosinophils, but no granulomas were detected (Figure, panel A). Juvenile sporangia measured 10–70 µm and contained a single nucleus with prominent nucleoli, granular cytoplasm, and well-defined cell walls (Figure, panel B). Immature (intermediate) sporangia measured 80–150 µm, contained several nuclei, and had granular cytoplasm and thicker cell walls. Mature sporangia measured up to 400 µm and contained hundreds of immature and mature endoconidia enveloped by thin cell walls (Figure, panel C). Some showed large pores (10–20 µm, best visualized on Grocott methenamine silver stain) in direct apposition with mature endoconidia (Figure, panel D).

**Figure:**
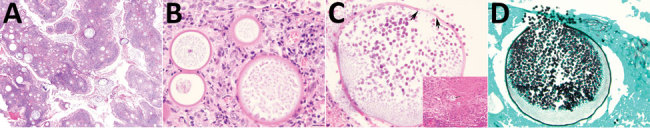
Histopathologic characteristics of rhinosporidiosis, Eastern Province, Rwanda, 2014–2016. A) At scanning magnification, multiple cystic structures (sporangia) of variable sizes are embedded in respiratory mucosa in a background of mononuclear inflammation (hematoxylin and eosin [H&E] stain; original magnification ×40). B) *R. seeberi* early juvenile sporangium, with a well-delimited cell wall, granular cytoplasm, and reddish nucleus with prominent nucleolus, characteristic of this stage (H&E stain; original magnification ×400). C) *R. seeberi* mature sporangium. Maturation of the endoconidia occurs from the periphery to the center of the cyst (H&E stain; original magnification ×600). Inset shows the spherules of coccidioides are smaller (up to 80 μm) and contain endospores of similar size, which appear as clear vacuoles (arrow) on stained sections (H&E stain; original magnification ×600). D) Both the cyst wall and endospores stain with the methenamine silver reaction, with discontinuity of the cyst wall at the upper pole, corresponding to the cyst pore zone (Grocott methenamine silver; original magnification ×400).

The main histologic differential diagnosis for rhinosporidiosis is coccidioidomycosis, a fungal disease only found in the dry areas of the Americas (endemic in California and Arizona, USA; Mexico; and Central and South America). The spherules of *Coccidioides immitis* measure 30–60 µm and contain uniform endospores of similar size (Figure, panel C, inset). In contrast, the sporangia of *R. seeberi* are much larger (100–400 µm) and contain variably sized endospores, ranging from small, hyperchromic forms to larger forms (7 µm).

We have documented a small cluster of nasal rhinosporidiosis occurring during a 2-year period in Rwanda. Rhinosporidiosis was first documented in Rwanda in the nasal passages of wild birds from Butare (then Astrida) in 1951, including a goose and a duck (*14*). The first 3 human cases were reported from Rwanda by Gigase and Kestelyn in 1993 as part of a 13-patient case series (*13*). Two Rwanda patients in this case series were part of a cohort of 12 patients in whom nasal rhinosporidiosis was diagnosed in Antwerp, Belgium. These cases were identified in a collection of ≈25,000 histologic specimens obtained from developing tropical countries during 1966–1988. The final case included in that study was diagnosed in 1986 in the Department of Ophthalmology at CHUK. That case was the only case diagnosed in 11 years among the ≈80,000 outpatients with ophthalmologic signs and symptoms seen at CHUK during that period (*13*). The remaining 10 patients were from Burundi (n = 2), Tanzania (n = 4), Zaire (now the Democratic Republic of the Congo) (n = 3), and Chad (n = 1). In contrast to larger case series identified in Africa (*6**–**13*), 2 of 3 previously reported cases (*13*) and all 4 of the more recent cases involved nasal rather than conjunctival disease. The contributing factors, if any, for onset of conjunctival versus nasal disease are not known and cannot be determined from our data.

Independent observations that rhinosporidiosis is epidemiologically associated with exposure to water, the placement of *R. seeberi* in a clade of aquatic parasites by molecular analysis, and the fact that watery substances facilitate the release of mature endoconidia from the sporangium (*14*) suggest that the natural niche of *R. seeberi* is water. Although the source of infection in the cohort we report cannot be determined from this study, the geographic clustering of cases suggests a possible reservoir of *R. seeberi* in the Eastern Province of Rwanda, given that all patients are native to that region and have no history of travel. Multiple water reservoirs and lakes exist in eastern Rwanda; Lake Muhazi is closest to Gatisbo District and Lake Cyambwe closest to Kirehe District, and the Kagera River runs nearby. In a previous study documenting 3 cases of rhinosporidioisis in Rwanda, the authors state that the cases “were native to the country, and as far as could be ascertained, native from places near the reference hospital” (*13*). However, no information about the place of origin or residence of those patients is available. 

The somewhat contemporaneous appearance of the cases we describe probably reflects the availability of diagnostic services, rather than a newly emerging disease. Until recently, anatomic pathology services were scarce in Rwanda. In October 2013, the anatomic pathology laboratory at CHUK restarted operations after many years, which is probably a major factor in detecting these cases; no other diagnostic test for the disease exists. A study by Jones et al. (*15*) found high spatial reporting bias for emerging infectious diseases, reflective of increased surveillance and infectious disease research in developed countries of Europe, North America, Australia, and parts of Asia compared with developing regions, including tropical Africa, despite the greater risk for emerging infectious disease in hotspots attributable to zoonotic pathogens from wildlife and vectorborne pathogens (*15*).

## Conclusions

We document a cluster of nasal rhinosporidiosis in eastern Rwanda. Clinicians and pathologists in Rwanda should be aware of this diagnosis because it might simulate a neoplasm and the disease is easily treatable. Although these findings might represent an emerging disease, the recent increase in availability of diagnostic pathology services probably played a major role in their identification.
